# Non-Linear EMG Parameters for Differential and Early Diagnostics of Parkinson’s Disease

**DOI:** 10.3389/fneur.2013.00135

**Published:** 2013-09-17

**Authors:** Alexander Y. Meigal, Saara M. Rissanen, Mika P. Tarvainen, Olavi Airaksinen, Markku Kankaanpää, Pasi A. Karjalainen

**Affiliations:** ^1^Institute of Advanced Biomedical Technologies, Petrozavodsk State University, Petrozavodsk, Russia; ^2^Department of Applied Physics, University of Eastern Finland, Kuopio, Finland; ^3^Department of Physical Medicine and Rehabilitation, Kuopio University Hospital, Kuopio, Finland; ^4^Department of Physical Medicine and Rehabilitation, Tampere University Hospital, Tampere, Finland

**Keywords:** Parkinson’s disease, electromyography, non-linear parameters, early diagnostics

## Abstract

The pre-clinical diagnostics is essential for management of Parkinson’s disease (PD). Although PD has been studied intensively in the last decades, the pre-clinical indicators of that motor disorder have yet to be established. Several approaches were proposed but the definitive method is still lacking. Here we report on the non-linear characteristics of surface electromyogram (sEMG) and tremor acceleration as a possible diagnostic tool, and, in prospective, as a predictor for PD. Following this approach we calculated such non-linear parameters of sEMG and accelerometer signal as correlation dimension, entropy, and determinism. We found that the non-linear parameters allowed discriminating some 85% of healthy controls from PD patients. Thus, this approach offers considerable potential for developing sEMG-based method for pre-clinical diagnostics of PD. However, non-linear parameters proved to be more reliable for the shaking form of PD, while diagnostics of the rigid form of PD using EMG remains an open question.

## Why to Improve Diagnostics of Parkinson’s Disease and Why to Do it Timely?

Parkinson’s disease (PD) is a progressive disorder which affects motor, higher mental and autonomic functions of the human organism. PD is the second most common, after Alzheimer’s disease, neurodegenerative disease. The incidence of PD in developed countries is estimated at about 1% in people older than 60 years and 4% in people over 80 years (1). PD is, in a sense, “non-lethal” pathology because it does not cause immediate fatal outcome. It can last for decades. Then, why is it so important to timely diagnose PD?

### Economical burden

Parkinson’s disease is still lethal due to motor deficits which cause falls. Also, PD implies burden of several kinds on the PD patients, their relatives, and society. The personal burden means dramatic decrease of the quality of the patient’s life due to motor (resting tremor, muscle rigidity, bradykinesia and/or akinesia, postural instability, and fatigue) and non-motor symptoms (constipation, impaired heart rate variability, depression, sleep disorders). These motor and non-motor symptoms may decrease person’s ability to work and thus may imply early retirement, restrictions on profession choice and, hence, the salary (2). Cost burden of PD patients also includes medication, care costs, insurance etc. Annual economical burden on one PD patient in the developed countries exceeds 6000–25000 USD (3).

### Differential diagnosis between various tremulous states

Parkinson’s disease diagnosis is highly uncertain in the early stages and only 70% of patients are correctly diagnosed with PD (4). PD is characterized by the symptoms which are shared with such similar, though still different, motor disorder as essential tremor (ET). There is a large body of papers on comparative studies of PD and ET (5). However, a diagnostic tool to differentiate these two pathologies is yet to be elaborated (6).

### Differential diagnosis between clinical forms of PD

PD is clinically not uniform and is presented by at least three clinical forms – tremulous-dominant, or shaking form, akinetic-rigid form and mixed form. The incidence of tremor-dominant and mixed (tremor plus akinesia/rigidity) type of PD is as much as 75% (7). Correspondingly, 10% of PD patients never have signs of resting tremor (8).

### Physiological tool

Parkinson’s disease may provide insight into such phenomena of the motor system as muscle tone, posture, gate and tremor, and yet enigmatic “motor commands,” and “motor programs.” For example, a characteristic spine bent “posture of beggar” in PD patients is analogous to the “tired ape” stance of astronauts/cosmonauts first described by Edwin Aldrin during Lunar mission (9) or to the “posture of embryo” under cold exposure (10).

### Long pre-clinical phase

Parkinson’s disease is characterized by a lengthy prodromal, which is known as either “pre-clinical” or “pre-diagnostic” phase (11, 12). The primary cause of PD is progressive loss of dopaminergic neurons in the compact part of *Substantia nigra*. When approximately 60% of these are lost, PD becomes clinically recognizable. This phase is believed to start long before emergence of clinical PD and then slowly progresses usually over 4.5 years (13, 14). Besides formidable challenge in early diagnosis, the prodromal period also presents unique opportunity in disease prevention or delay in PD onset (15). As such, “disease modification,” “slowing down,” or “neuroprotection” are emerging terms in respect with PD (15). Approximately 10% of subjects over 60 years are in the “pre-diagnostic” phase of PD according to neuropathological reports (16). Therefore, predating the diagnosis of PD and identifying subjects at-risk is an important goal for research aimed to postpone the onset of PD by neuroprotective therapy (17, 18). Thus, the ultimate goal of the early diagnosis of PD would be to switch from medical treatment to disease management. Becker et al. (17) suggest two approaches in order to reach that goal. The first one would be to detect subjects with risk factors for PD using currently available tests, such as scanning *Substantia nigra* using functional magnetic resonance imaging (fMRI), ultrasound, and genetic identification. The other approach would be to detect PD patients at the very initial phase of the disease when only few non-motor or “soft” motor symptoms are detectable.

Thus, pre-clinical detection of PD seems to be an important goal because even subtle motor or non-motor pre-clinical abnormalities may serve as “predictors” for further PD. Such predictive study would help identifying the subjects at-risk of future PD, to start earlier anti-PD treatment, and to develop effective neuroprotective treatment strategies (18). Here, we report on current approaches in early diagnostics and differential diagnosis of PD with special stress on the non-linear parameters of interference surface electromyography (sEMG) signal.

## Current Approaches to Early Diagnosis of PD

Patients with PD have several symptoms other than motor ones (the non-motor symptoms). Few of them have been proposed for early detection of PD. Among them are: (1) olfactory disorder; (2) sleep disorder; (3) autonomic features (heart rate variability and constipation); (4) color vision disorders; and (5) so-called “soft” signs of PD such as reaction time slowing, depression, mid-life obesity, and non-specific pain in joints (19). These disorders are associated with functioning of dopaminergic synapses and may reflect progression of dopamine deficit from brainstem to neocortex, as proposed by Braak (20). Accumulating evidence suggests that the above mentioned symptoms develop namely during a long prodromal period of PD (21).

Olfaction is impaired first in PD due to affection of the olfactory bulb, thus forming stage I of PD (20). Smell dysfunctions, such as hyposmia, anosmia, impaired odor detection, discrimination, or identification affects more than 80% of PD patients (19). Visual dysfunction is suggested to be caused by a dopaminergic deficit of the retinal neurons (16). Indeed, dopaminergic therapy improves visual impairment (22). Rapid eye movement (REM) sleep behavior disorder (RBD) is a prodromal marker for PD, and it is characterized by the loss of normal skeletal muscle tone during REM sleep in association with increased EMG of limb and chin muscles, excessive limb jerking and dream mentation (14, 23). Occurrence of RBD in PD patients varies from 15 to 47% (23). Interestingly, olfaction, REM and visual disorders are usually synchronized with each other thus forming a unique set of associated symptoms (24).

Autonomic symptoms, such as constipation and heart rate variability, and affective symptoms (depression, phobia) are also candidates for early stage PD diagnostics (14, 17, 19, 25). Dopamine loss may also produce subtle (“soft”) subjective motor complaints, such as slowed reaction time, imbalance, changes in handwriting, speech, or reduced arm motion. In particular, impairment of orofacial motor functions (articulation, phonation, prosody) may lead to speech defectiveness due to weakness of tongue and lips musculature (26). It has been demonstrated that 78% of early untreated PD subjects indicate some form of vocal impairment (27). Impairment of handwriting, Archimedes spiral drawing, and hand tapping may also indicate for PD. Advanced analysis of spiral metrics presented high correlation with UPDRS (Unified PD Rating Scale, part III) (28). Variation of hand rhythmic tapping was increased in tremor predominant group of PD patients (29). These symptoms may be identified years before the diagnosis of PD is made (17).

Over the last years, single photon emission computed tomography (SPECT), positron emission tomography (PET), fMRI and transcranial sonography are widely used to assess dopaminergic function in PD patients, their relatives, and healthy controls (17). However, their diagnostic precision is still far from satisfactory (13). For example, up to 15% of subjects with normal imaging findings have clinically evident PD, and vice versa, decreased dopamine content in the *Basal ganglia* seen on MRI is often associated with neurodegenerative diseases other than PD (dementia with Lewy bodies, multiply sclerosis atrophy) (30). Also, methods based on nuclear medicine and ultrasound are not appropriate for population-based studies due to their high costs, and insufficient availability (17).

In a whole, definitive evidence for a PD-sensitive diagnostic tool is lacking. Combination of the above mentioned methods would probably be the best current solution for pre-clinical diagnosis of PD. The decisive diagnosis of PD is still post-mortem. All in all, current methods help to diagnose only 70–80% of the PD cases (17, 31), which is not satisfactory. It corresponds with misdiagnosis of PD estimated as 20–30% (8, 30). Novel biomarkers for PD must be presented.

## EMG as a Potential Early Marker for PD

Electromyography (EMG) helps investigating the central nervous system (CNS) because it reflects the activity of the spinal motoneurons due to motor units (MUs). We seek to provide a readable method to diagnose PD based on EMG. In ideal, such method would also be helpful to detect PD either at early stage or even pre-clinically. Several studies have reported that MUs in PD patients discharge with alternating shorter and longer interspike intervals (doublets or triplets). This pattern of activity is strikingly different from stationary activity of MUs under normal muscle tone (32–34). Nonetheless, the doublet pattern is not specific for PD and can be seen under other normal and pathological conditions. For example, doublets are seen in humans at the onset of strong and ballistic movements (35), during whole-body heating (36), and after dynamic training (37). Doublets are common in neuropathies (38) and amyotrophic lateral sclerosis (39). Also, MU action potentials are usually recorded using needle electrodes, i.e., intramuscularly. This is uncomfortable for the patient and requires antiseptic measures.

There has been an attempt to combine EMG with thermal interventions as a provoking factor for PD symptoms. Cold was reported to intensify tremor in PD patients, especially with tremor-dominated form, while heat reportedly attenuated muscle rigidity (40). Nonetheless, cold exposure is likely not reliable for early diagnostics of PD due to its apparent unpleasantness and procedure requirements. Cold also can provoke chill and cold shivering, that would require further analysis to distinguish it from PD tremor.

In contrast to needle EMG, surface EMG (sEMG) is non-invasive (less discomfort and risk of infection), more stable in respect with electrode position (more repeatable), and cost-efficient. sEMG has been extensively used to examine motor function and movement disorders in humans and it is believed to provide relevant information on neuromuscular strategies (41). Spectral-based analysis methods have diagnostic value for PD (42–46). However, no consensus exists about applicability of sEMG to PD diagnosis because conventional linear parameters are still lacking to provide definitive difference between the PD and healthy controls.

The morphology-based analysis has shown promising results in discriminating PD and healthy controls. The method is based on the histogram and crossing rate analysis of sEMG signals (44). sEMG kurtosis, a parameter based on higher order statistics, is reportedly increased in PD patients. This might reflect increased number of spikes due to increased synchronicity of MU firing (47). Turn/amplitude analysis (TAA) of sEMG, a method that couples number of turns on sEMG (reversal of sEMG signal direction with amplitude >100 μV) with average sEMG amplitude, is still largely used to discriminate between neurogenic and myogenic affections (48, 49). To the best of our knowledge, TAA yet was not applied to study PD.

Previous works have reported that sEMG waveform can better be modeled as an output of a non-linear dynamic system, rather than a stochastic output of a white-noise driven linear system (38). Non-linearity is a hallmark of complex dynamic systems (50, 51). As a non-linear signal, sEMG displays chaotic behavior, i.e., its time series (1) evolves over the time, (2) depends on the initial state, and (3) is fractal in the terms of dimensionality (52). Thus, as a non-linear signal, sEMG can be characterized by the state of deterministic chaos (53). Therefore, sEMG might give clues to describe dynamics of the neuronal circuits in the terms of regularity, predictability, and complexity (54). Indeed, it has been recently found that non-linear parameters, such as approximate entropy (ApEn), percent of determinism based on recurrence quantification analysis (RQA), and dimensionality based on fractal analysis are highly sensitive for hidden rhythms on sEMG in subjects under fatigue and condition of increased MU synchronization (43, 55–58).

sEMG in PD patients is known to be rich in regular clusters (grouping) at the characteristic tremor frequencies (4–6 Hz) due to increased synchronization of MU (59). Also, determinism of sEMG in PD patients at rest was higher than during voluntary isometric contraction (42). Acceleration signal has also been studied in PD patients using both linear and non-linear parameters (59, 60). These findings led us to ask whether either readily visible or “hidden” rhythms in sEMG contribute to its non-linear features and thus yield a difference between PD patients and healthy controls. If this hypothesis holds, PD patients might present a more regular time-dependent structure of sEMG and acceleration time series, while healthy subjects – a less regular and more complex signal. Also, PD patients with lower UPDRS score might present less regular signal, either sEMG or acceleration. We compared a variety of novel non-linear parameters with the classic linear parameters of sEMG and acceleration signal between PD patients with various UPDRS scores, and found that this hypothesis holds true (47, 61). The results and conclusions are presented in the following sections.

## Linear and Non-Linear Variables of sEMG and Acceleration in PD Patients

### sEMG signal

This subsection deals with our previous study (47), in which we studied PD patients (*n* = 30) and two healthy control groups of different age – young (*n* = 20) and old (*n* = 20). sEMG was recorded bilaterally in the upright stance from biceps brachii muscles under elbow flexion. The loading conditions were 0, 1, and 2 kg respectively. Complexity and regularity of SEMG was analyzed by various methods of non-linear time series analysis, including sample entropy (SampEn), correlation dimension (CD), percent of determinism (DET%), and recurrence rate (REC%) based on RQA. The amplitude of sEMG was defined as the root mean square (RMS) value and median frequency (MDF) was also determined for analysis.

The major finding of our studies was that non-linear parameters of sEMG signal in the PD group significantly differed from the ones in the healthy control groups (47). In particular, %REC and %DET values of SEMG were significantly higher in the PD group, while SampEn and CD were lower in comparison to old and young controls (Table 1). Instead, such traditional parameters as RMS and MDF did not differ between groups (Table 1).

**Table 1:** **The linear and non-linear SEMG parameters of PD patients and healthy old and young control subjects**.

Group	No load		1 kg load		2 kg load	
	Right	Left	Right	Left	Right	Left
**RMS (μV)**						
PD	48.42 ± 29.17	41.09 ± 29.50	81.90 ± 48.22	61.91 ± 35.01*^#^	107.63 ± 58.44	88.6 ± 48.7*^#^
Old	40.81 ± 20.91	41.28 ± 18.83	68.04 ± 32.26	70.62 ± 30.83	96.52 ± 48.98	100.32 ± 44.4
Young	42.83 ± 22.87	51.88 ± 26.08	72.83 ± 27.09	98.27 ± 48.34	108.15 ± 41.86	139.41 ± 60.77
**MDF (Hz)**						
PD	55.10 ± 15.28	57.10 ± 10.57	60.70 ± 12.17**	62.19 ± 10.78*	59.13 ± 11.81*	58.46 ± 7.56
Old	56.20 ± 7.69	59.86 ± 10.86	56.00 ± 6.63	60.57 ± 10.97	55.47 ± 5.01	59.54 ± 10.74^§^
Young	50.68 ± 5.98	53.10 ± 8.41	51.63 ± 5.57	53.07 ± 7.23	51.90 ± 5.48	52.14 ± 6.09
**PERCENTAGE OF RECURRENCE (%REC)**						
PD	21.4 ± 18.9**^##^	16.5 ± 17.6*^#^	17.6 ± 15.5**^#^	15.5 ± 19.1	16.7 ± 16.9*^#^	11.8 ± 11.9
Old	7.3 ± 4.7	7.8 ± 3.7	6.8 ± 2.2	10.3 ± 7.8	6.6 ± 2.6	10.3 ± 7.8
Young	7.4 ± 4.3	7.7 ± 4.3	8.9 ± 4.3	8.4 ± 4.2	7.7 ± 3.4	8.3 ± 3.8
**PERCENTAGE OF DETERMINISM (%DET)**						
PD	32.6 ± 33.5**^#^	24.8 ± 27.8*	26.20 ± 27.6*	22.3 ± 26.3	28.5 ± 27.9*	20.7 ± 22.5
Old	11.6 ± 7.1	9.7 ± 5.1	12.3 ± 6.4	12.9 ± 7.2	12.1 ± 6.5	12.9 ± 7.2
Young	17.0 ± 7.4	15.8 ± 9.4	19.0 ± 8.0	18.3 ± 8.4	19.3 ± 8.8	20.5 ± 9.4
**SAMPLE ENTROPY (SAMPEN)**						
PD	0.93 ± 0.35**	1.03 ± 0.31*	1.05 ± 0.35	1.08 ± 0.37	1.01 ± 0.35	1.10 ± 0.24
Old	1.17 ± 0.11	1.20 ± 0.11	1.17 ± 0.10	1.21 ± 0.14^§^	1.15 ± 0.10	1.17 ± 0.14^§§^
Young	1.02 ± 0.11	1.00 ± 0.14	1.02 ± 0.13	1.02 ± 0.15	0.99 ± 0.14	0.98 ± 0.14
**CORRELATION DIMENSION (CD)**						
PD	4.86 ± 2.51**^##^	5.63 ± 2.33*	5.28 ± 2.35**^#^	6.05 ± 2.42	5.59 ± 2.41*^#^	6.26 ± 1.97
Old	6.92 ± 1.15	6.97 ± 0.73	7.10 ± 0.62	6.54 ± 1.25	6.98 ± 0.82	7.18 ± 0.63
Young	6.77 ± 1.07	6.76 ± 0.98	6.61 ± 0.83	6.54 ± 1.08	6.77 ± 0.86	6.72 ± 0.80

The data was analyzed in study (50).

*PD to young (p < 0.05); **PD to young (p < 0.01); ^#^PD to old (p < 0.05); ^##^PD to old (p < 0.01), ^§^old to young (p < 0.05).

**Figure 1:**
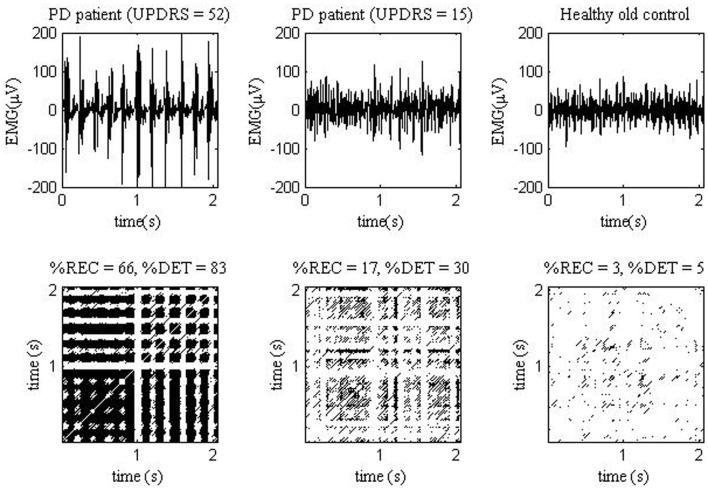
**EMG signals (top) measured from one PD patient with UPDRS = 52 (left), one PD patient with UPDRS = 15 (middle), and one healthy old control (right)**. The EMG measurements were analyzed in study (47). The corresponding EMG recurrence plots (bottom).

Decreased CD of sEMG in the PD group may indicate increased self-similarity of the myoelectrical signal over time (18) and hence, lowered complexity of the underlying neural network. Decreased sample entropy of sEMG signal in the PD group may reflect higher regularity of sEMG. These findings are in line with earlier studies, which have documented higher sensitivity of %DET and entropy to motor unit synchronization, than spectral frequency characteristics (44, 56, 57, 59, 60). High %DET reflects abundant wave features in sEMG, either readily visible by eye or quasi-waves during, e.g., increased motor unit synchronization (44). Clustering of MU action potentials was the characteristic of raw sEMG in many our PD patients. Interestingly, %DET also was increased in some patients with visually stationary sEMG. Figure 1 shows sEMG signals and recurrence plots obtained from PD patient with higher (with distinct sEMG clustering) and lower UPDRS score (with stationary-looking sEMG), and from a healthy older subject.

Thus the “continuum” of UPDRS score from lower to higher values may correspond with “continuum” of sEMG parameters. In fact, we have found that such novel sEMG parameters, as %REC, and %DET were significantly correlated with UPDRS score (*R* = 0.47–0.71) (47). Thus, PD patients with less expressed sEMG clustering indeed present less regular signal. Most of novel sEMG characteristics correlated also with finger tapping scores (*R* = 0.54–0.66) (47). Correlation values were the most significant in the state without additional loading, and they decreased when loading increased. This probably indicates emergence of “regular normal” postural muscle tonus, which erased the difference between the groups. In a sense, under loading sEMG of PD patients became more “normal.”

Thus, sEMG signal in PD is less complex, more predictable and regular. It means that rhythmic activity takes place in the spinal cord, resulting in more or less obvious clustering of sEMG. From the physiological point of view, these data reflect increased synchronization of MU activity or increased clustering coefficient of signal generator (the spinal neuron circuitry) (56). It could also well be that the spinal cord rather relays this periodicity from the upper levels of CNS, than generates it itself. In fact, high regularity of sEMG signal in PD is associated with rhythmic oscillatory activity in the CNS (62). The 4–7 Hz parkinsonian tremor may be associated with increased θ-rhythm (4–7 Hz) in EEG, thus indicating general slowing of oscillatory brain activity with the time course of PD (63).

### Acceleration signal

In another previous study by our group (61), we measured accelerometer (ACC) characteristics of tremor: (1) the amplitude of ACC, computed as the RMS value of the signal; (2) the frequency (F) corresponding to the maximum power in acceleration spectrum; (3) the coherence spectrum (Coh) between sEMG and acceleration signals, which describes the similarities in the power spectra of two time series. Time-dependent structure of ACC was analyzed using SampEn, CD, DET% and REC% (61). %DET of the acceleration signal was much higher in the PD patients (mean 50%) when compared to young and old (mean 11–13%) controls while ApEn of tremor signal is 15–22% lower in PD patients than in healthy controls (61). Thus, tremor is more regular in PD in comparison with healthy controls, that is in line with earlier studies (59, 60).

Acceleration signal demonstrated a very much the same correlation with loads, UPDRS, and motor symptoms as the sEMG signal (61). However, there was much less “merging” of non-linear parameters values under loading, probably due to different origins of sEMG and acceleration signals.

**Figure 2:**
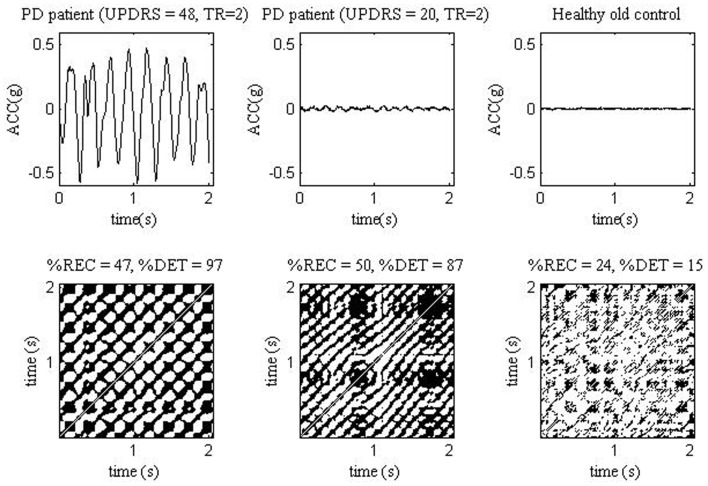
**ACC signals (top) measured from one PD patient with UPDRS = 48 and rest tremor TR = 2 (left), one PD patient with UPDRS = 20 and TR = 2 (middle) and one healthy old control (right)**. The ACC measurements were analyzed in study (61). The corresponding ACC recurrence plots (bottom). TR, tremor score.

%DET, SampEn, and amplitude of acceleration signal, though less than that of sEMG, correlated with the UPDRS score (*R* = 0.47–0.52) and finger tapping (*R* = 0.32–0.46) (61). Thus, acceleration signals in the PD group contain large portions of recurrent fragments. This evidences a highly deterministic, time-dependent structure of tremor in the PD group. Moreover, tremor in PD patients is more deterministic, the larger is the UPDRS score. It means that tremor under PD progression not only grows in its amplitude, but becomes more regular (61). Figure 2 shows ACC signals and recurrence plots obtained from PD patient with higher and lower UPDRS score, and from a healthy older subject.

No major difference in sEMG and acceleration characteristics was found between old and young controls (47, 61). That is in a line with a postulation that healthy aging does not lead to major changes in postural tremor (60). According to our data only peak frequency and %REC were smaller in older subjects, while SampEn, cross-SampEn, and CD were slightly greater when compared to younger subjects. Similarly, in the studies of Vaillancourt et al. (46) and Sturman et al. (60), ApEn of acceleration signal was shown to be greater, although insignificantly, in older healthy subjects compared to the younger subjects, especially at lower loadings. The same tendency, also insignificant, we observed for the EMG parameters (47).

## Other Signals

There are few studies on the non-linear properties of signals in PD other than sEMG, i.e., EEG and acoustic (voice). Their results mainly showed that the EEG of PD patients is characterized by higher entropy (64) and CD (59). Such higher complexity may reflect reduced disfacilitation of competing motor programs, resulting in a larger number of simultaneously active neural networks (65). The fractal dimension of the acoustic signal of sustained vowel production is reduced in PD patients as compared to the respective controls (66).

## Prospective and Pitfalls

We believe that non-linear parameters of sEMG have potential in differential diagnosis of PD and it is promising for early pre-clinical diagnostics of PD. In our recent studies (67, 68), different EMG and acceleration signal features, including non-linear, were extracted and used to form high dimensional feature vectors for the cluster analysis of subjects. According to clustering results, one cluster contained 90% of the healthy controls and two other clusters 76% of PD patients (67). This can be regarded as a promising result when compared to SPECT or clinical diagnosis (46, 69). EMG burst characteristics were also analyzed during flexion and extension movements in the study of Rissanen et al. (68). The discrimination rates between patients with PD and healthy controls obtained in this study (73%/82% in flexion and 80%/87% in extension) depict a rather high sensitivity/specificity of the method. However, at best the sEMG/acceleration method discriminates as much as 80% of PD patients from healthy controls that is yet far from desirable 100%. Two major reasons may prevent sEMG of reaching a more precise discrimination.

### PD non-uniformity

First, it could well be so that 10–20% of PD patients which cannot be distinguished from healthy controls by non-linear parameters, belong to the patients who never have signs of resting tremor (akinetic-rigid form). In fact, in our study, the portion of PD patients without tremor was 10%. sEMG from the rigid muscle lacks rhythms, which are characteristic of the tremor, due to asynchronous stationary discharges of MU. Therefore, muscle tone from the rigid muscle is non-distinguishable from regular postural muscle tone (33). As such, new tools must be elaborated and tried to detect, namely rigidity.

### Methodological limitations

Second, much of PD diagnoses are still false (4, 14). In our study, it may be so that some patients actually did not have PD and thus they might contribute to the overlapping of PD patients and healthy controls. Vice versa, some subjects considered as healthy, might actually be not. In theory, it could well be that some of older healthy controls had the pre-clinical stage of PD. Similarly, some younger healthy controls could have had ET.

In ideal, to find a discriminating characteristic, one would compare a group of PD patients with true diagnose and a group of true healthy persons. As for now, it is difficult to arrange so. We still have to rely on the clinical diagnosis, UPDRS, or SPECT, at best. Also, the lowest UPDRS score in our study was as little as 14. It would be interesting to examine, whether novel sEMG parameters are sensitive to even lower UPDRS scores.

To overcome these methodological pitfalls, one should consider designing a longitudinal study of PD. Namely, sEMG and acceleration could be recorded from a cohort of older healthy subjects over the age of 60 years. Then, these subjects could be investigated in respect with non-linear parameters every 1–2 years. Statistically, 10% of them are on their pre-clinical stage of PD (16), and eventually some of them will develop the clinical form. In that case, the early changes on sEMG or acceleration could be detected. Also, there are few genes that have a significant impact on the development of Parkinson’s disease (70). People with these genes and the relatives of PD patients could be tested for presumably subtle changes in sEMG parameters.

## Conclusion

The novel sEMG parameters have potential in the pre-clinical diagnosis of PD due to their relatively high discrimination power, cost efficiency and high throughput.

## Conflict of Interest Statement

The research was conducted in the absence of any commercial or financial relationships that could be construed as a potential conflict of interest.
